# Lack of association between putative transporter gene polymorphisms in *Plasmodium falciparum *and chloroquine resistance in imported malaria isolates from Africa

**DOI:** 10.1186/1475-2875-5-24

**Published:** 2006-03-28

**Authors:** Sandrine Cojean, Alain Noël, Dimitri Garnier, Véronique Hubert, Jacques Le Bras, Rémy Durand

**Affiliations:** 1 Centre National de Référence pour la Chimiosensibilité du Paludisme, APHP, Hôpital Bichat-Claude Bernard, Paris, France; 2 Laboratoire de Biologie Animale et Parasitaire, Université Descartes Paris 5, Paris, France; 3 Laboratoire de Parasitologie Mycologie, Hôpital Avicenne, 125 rue de Stalingrad, 93009 Bobigny Cedex, and Université Paris 13, EA 3406, Bobigny, France

## Abstract

**Background:**

*Plasmodium falciparum *drug resistance represents a major health problem in malaria endemic countries. The mechanisms of resistance are not fully elucidated. Recently, an association between putative transporter gene polymorphisms and in vitro response to chloroquine (CQ) and quinine has been reported in culture-adapted, cloned isolates from various geographical origins. However, this was not confirmed in another study performed on isolates from a defined region in Thailand.

**Methods:**

This study tried to find an association between putative transporters gene polymorphisms with in vitro response to CQ and *pfcrt *genotype in isolates originating from various African countries. To avoid biases of parasites adaptation in culture, fresh isolates obtained from symptomatic, malaria-infected travellers returning from Africa to France were used. Monoclonal isolates included in the study were selected using a *msp-2 *fragment analysis method. In vitro susceptibility to CQ, single nucleotide polymorphisms and microsatellite polymorphisms in *pfcrt*, *pfmdr1 *and six putative transporter genes were established in 27 isolates and three reference strains.

**Results:**

Polymorphism of *pfcrt *at positions 76 and 220 showed a significant association with in vitro chloroquine resistance (*P *< .02 and *P *< .05 respectively). Polymorphism of *pfmdr1 *at position 86 showed an equally significant association with in vitro chloroquine response (*P *< .05). No association was found between SNPs or microsatellite polymorphisms of putative transporter genes and in vitro CQR or *pfcrt *genotype in imported malaria isolates from Africa.

**Conclusion:**

The previously described association between putative transporter gene polymorphisms and in vitro response to chloroquine (CQ) was not confirmed in the present study.

## Background

*Plasmodium falciparum *malaria remains one of the major causes of morbidity and mortality in sub-Saharan Africa, leading each year to the death of an estimated number of 1–2.7 million individuals, mostly children [1]. Chloroquine (CQ) resistance of *P. falciparum *represents today a major health care problem in malaria endemic countries [2]. PfCRT, a member of the drug/metabolite transporter superfamily, was demonstrated to play a central role in the resistance of *P. falciparum *to CQ [3-6]. The identification of PfCRT and the discovery of its role in CQR were a step by step work. Using a genetic cross, the Wellems'group demonstrated that a 36 kb locus in chromosome 7 was linked to CQR [7,8]. Initially a first candidate gene, *cg2*, comprised in that locus and thus associated to CQR, was considered as responsible for CQR [8-10]. However further studies refuted the hypothesis [11] and proposed another gene equally comprised in that locus, *pfcrt*, as the real, though not necessarily unique, cause for CQR. The story of *cg2 *is a reminder that the apparent association of a molecular marker with in vitro drug resistance is not synonymous of causality. Such associations must be verified on numerous isolates originating from various geographical areas and further molecular studies are required to assess the involvement of the candidate genes in drug resistance. Other molecules, as Pgh1, a member of the ABC transporter superfamily encoded by *pfmdr1*, may contribute to CQR [12,13]. However, the implication of *pfmdr1 *in CQR remains uncertain [14-16]. The mutation at *pfmdr1 *Y86 may compensate deleterious *pfcrt *mutations or have a role in CQR by itself [17-19].

With the availability of the *P. falciparum *genome, a new era in the identification of genes potentially involved in antimalarials resistance has opened. Mu *et al*. reported the association of putative transporter gene polymorphisms with in vitro response to CQ and quinine [20]. Among 49 genes encoding putative transporters identified in *P. falciparum*, Mu *et al*. found nine transporters other than products of *pfcrt *and *pfmdr1*, including mainly putative ABC transporters, which where associated with *in vitro *CQR and/or quinine resistance. Six new genes were associated with the CQ response from Southeast Asian isolates (*G2, G25, G47, G49, G54, G70*) and three genes were associated with the CQ response from African isolates (*G7*, *G30*, *G55*). A limitation of that study was that the IC_50_s and single nucleotide polymorphisms (SNPs) determinations were performed on culture-adapted cloned isolates, which could lead to biased results due to accumulated mutations selected by in vitro conditions. Anderson et al. studied isolates from a large number of patients from a single clinic located on the Thai-Burma border [21] and did not observe any significant association between in vitro CQ response and the polymorphisms of the putative transporters which had been described as strongly associated with CQR in the original study. In particular, they did not find an association between eight of the nine studied genes and response to eight different antimalarial drugs, though they reported an association between *G7 *gene polymorphism and in vitro artesunate response. However, these authors did not exclude that some of the studied loci may be associated with CQR in other regions of the world.

The present study tried to reproduce Mu's data in isolates originating from various African countries. To avoid biases of parasites adaptation in culture, fresh isolates obtained from symptomatic, malaria-infected travellers returning from Africa to France were used. Furthermore, to avoid errors due to multiple infections, monoclonal isolates were selected by a *msp-2 *fragment analysis method [22]. No association was found between putative transporter genes polymorphisms and in vitro CQ susceptibility in imported malaria isolates from Africa.

## Materials and methods

### Clinical isolates

Isolates were obtained from patients admitted to a Department of Infectious and Tropical Diseases, an Emergency or Intensive Care Unit of a metropolitan French hospital with a diagnosis of *P. falciparum *malaria, between January 1998 and December 2004. All patients had returned from various countries of Africa (Table 1).

**Table 1:** Amino acid residue 76 of the *pfcrt *gene and chloroquine response phenotype of 27 *P. falciparum *isolates and 3 reference strains.

Isolates^a^	CI_50_^b^CQ (nM)	*pfcrt *K76T
Ivory Coast 1	5.3	K
Cameroon 1	13.5	K
Senegal 1	16.8	K
Senegal 2	18.8	K
Senegal 3	23.6	K
Nigeria 1	24.8	K
Congo 1	25.6	**T**
Madagascar 1	27.3	K
Senegal 4	33.0	K
Madagascar 2	43.4	K
Senegal 5	44.2	**T**
Mauritania 1	46.6	K
Ivory Coast 2	47.6	**T**
Madagascar 3	50.0	K
Cameroon 1	52.9	**T**
Mali 1	53.1	**T**
Togo 1	60.4	**T**
the Comoros Island 1	74.5	K
Mali 2	79.6	**T**
Ivory Coast 3	88.2	**T**
Ivory Coast 4	99.6	**T**
Benin 1	100.0	**T**
Ivory Coast 5	100.0	**T**
Mali 3	121.0	**T**
Cameroon 3	169.0	**T**
Togo 2	173.0	**T**
Kenya 1	219.0	**T**

Africa 3D7 strain	18.5	K
Indochina W2 strain	370.0	**T**
Cameroon FCM29 strain	1105.0	**T**

^a^Isolates are designated by the name of their country of origin followed by a number.

^b^Fifty percent inhibitory concentration values : resistance value to chloroquine (CQ) corresponds to IC_50_>100 nM.

Three reference strains adapted in culture were used: the chloroquine sensitive 3D7 line and the chloroquine resistant W2 and FCM29 lines; 3D7 originated from Africa, W2 from Indochina and FCM29 from Senegal.

### Malaria diagnosis

Giemsa-stained thin and thick blood smears were observed by light microscopy at a magnification of x1,000. Microscopy was independently performed by two experienced technicians, each time comparing their results with the other. Cases due to species associations including *P. falciparum *were not considered in the analysis.

### Determination of in vitro susceptibility to chloroquine

The in vitro susceptibility to chloroquine of each isolate was determined on parasitized red blood cells taken from the patient, using the semi-microtest method [23]. The 50% inhibitory concentration (IC_50_) using tritiated hypoxanthine uptake was calculated by non-linear regression analysis.

### Parasite DNA preparation

DNA was extracted from clinical isolates using QIAamp^® ^mini kit (Qiagen France S.A.), following manufacturer protocols.

### Clonal analysis

The number and the proportion of genotypes within isolates were determined for each patient using a previously published fragment analysis method [22]. Briefly, the method was based on the polymorphism of the gene encoding merozoite surface protein 2. The amplification of a part of merozoite surface protein-2 (*msp-2*) gene by polymerase chain reaction (PCR) with a fluorescent primer, followed by capillary gel electrophoresis, was used to discriminate alleles of different sizes. Each genotype was characterized by the size and the area under the curve of the peak corresponding to its *msp-2 *PCR products measured during the capillary electrophoresis. This methodology allowed to detect all clones accounting for more than 1% of the whole. The description of each isolate included the number of *msp-2 *genotypes, the size of the corresponding PCR products and the proportion of each genotype (given in percentage) within the isolate. Isolates showing only one single peak on the capillary gel electrophoresis were considered as being monoclonal and selected for the study.

### Genotyping of pfcrt, pfmdr1 and putative transporter genes

The *pfcrt *domains were amplified using the following conditions: 0.3 μM of each primer (Table 2), 200 μM of dNTPs, buffer (50 mM KCl, 10 mM Tris-HCl, pH 8.3, 1 mM MgCl_2_) and 2,5 U of *thermus aquaticus *DNA polymerase (AmpliTaq GoldTM, Applied Biosystems, APPLERA France S.A.). The samples were incubated for five minutes at 94°C prior to 40 cycles (94°C x 40 seconds, 59°C x 40 seconds and 72°C x 40 seconds), followed by one minute at 72°C. PCR products spanning codon *pfcrt *76 were analysed by restriction fragment length polymorphism (RFLP). Eight μL of PCR products were digested by the restriction enzyme Apo I (New England Biolabs, Beverly) at 50°C. RFLP products were electrophoresed on 1,5 % agarose gels and visualized under UV after staining with ethidium bromide.

**Table 2:** Oligonucleotide sequences and hybridization temperatures (HT) used for genotyping transporter polymorphisms.

Gene^a^	Predicted products	Position	AA change	Primer (sense)	Primer (antisense)	HT (°C)
*pfcrt*	Putative transporter	76	K-T	5'-TTTAGGTGGAGGTTCTTG-TC-3'	5'-ATAAAGTTGTGAGTTTCGGA-3'	59
		220	A-S	5'-TTATACAATTATCTCGAAGCAG-3'	5'-CATGTTTGAAAAGCATACAGGC-3'	59
*pfmdr1*	ABC transporter	86	N-Y	5'-AGAGAAAAAAGATGGTAACCTCAG-3'	5'-ACC-ACAAACATAAATTAACGG-3'	51
*G7 *(PF13-0271)	ABC transporter	1390	&1	5'-GCTAAAGAAAAGGATCCGAACA-3'	5'-CAACCTTTTCTCCATTTTCAAT-3'	50
*G25 *(PF14-0679)	Sulfate permease	Intron	G-A	5'-TATGGGAGGTGCTGAATGTG-3'	5'-TCGTTATCTTCGAAATTGTAGCC-3'	61
*G30 *(PF14-0292)	GTPase	Intron	C-G	5'-AATTGCAAAGGGAAGGAAGG-3'	5'-TTGGGTACACGCACGTTAAG-3'	61
*G49 *(PF08-0078)	ABC/ATPase	146	Q-E	5'-GGAAGGCATTATAGCAAACC-3'	5'-AGTCGTTTTCCCACATCCA-3'	56
		1046	L-I	5'-TGAACTTATTGAGACCGGTGAA-3'	5'-ACGCACTTTTCACCTCCA-3'	56
		1116	L-I	5'-TGAACTTATTGAGACCGGTGAA-3'	5'-ACGCACTTTTCACCTCCA-3'	56
*G54 *(PF14-0260)	Membrane protein	141	Y-Y	5'-TTGGATCAGACATTACCATC-3'	5'-GGATATTGTTCCTCAAGCTCCT-3'	56
		144	T-T	5'-TTGGATCAGACATTACCATC-3'	5'-GGATATTGTTCCTCAAGCTCCT-3'	56
*G55 *(PF14-0133)	ABC transporter	Intron	&2	5'-ATAATGCATACATAACCTTACC-3'	5'-TCCCATTTATGTAATATGAAC-3'	51
*G70 *(PFL0620c)	Choline transporter	105	E-K	5'-TTGATGCGTGTGTATTGATA-3'	5'-ATGTGAACCACCTTCTGGA-3'	56

^a^PlasmoDB (version 4) gene identifiers are shown in brackets

&1 represents a trinucleotide insertion

&2: microsatellite polymorphism located 700 bp downstream of the stop codon

PCR products spanning codon *pfcrt *220 and intron 4 associated microsatellite were purified using a QIAquick^® ^PCR Purification Kit (QIAgen, France S.A.) and sequenced. The sequencing reaction was realized using an ABI Prism^® ^Big Dye Terminator Cycle sequencing kit (Applied Biosystems, APPLERA France S.A.) following the manufacturer protocols. The fluorescent products were sequenced in an ABI Prism^® ^3100 Genetic Analyser (Applied Biosystems, APPLERA France S.A.) and examined with Chromas software.

*Pfmdr1 *domain was amplified as previously described [14]. Eight microliters of PCR products were digested with NspI (New England Biolabs, Beverly) at 37°C. RFLP products were processed as described above.

Amplifications of a part of the seven putative transporter genes spanning point mutations and microsatellites were performed using the following conditions : 0.3 μM of each primer (Table 2), 200 μM of dNTPs, buffer (50 mM KCl, 10 mM Tris-HCl, pH 8,3, 1 mM MgCl_2_) and 2,5 U of *thermus aquaticus *DNA polymerase (AmpliTaq GoldTM, Applied Biosystems, APPLERA France S.A.). The samples were incubated for five minutes at 94°C prior 40 cycles (94°C × 40seconds, hybridization temperature x 40 seconds and 72°C x 40 seconds) (see Table 2) followed by one minute at 72°C. PCR products underwent DNA sequencing as described above.

### Data analysis

The data were entered into a database in Excel 2003 and statistical analysis was performed using Epi Info^®^, version 3.3 (Centers for Disease Control, Atlanta, USA 2004). The comparisons between polymorphisms (SNPs) and in vitro resistance were done using Student test. P-values <0.05 were considered significant.

## Results

### Isolates and sensitivity testing

During the study period, 330 isolates had successful in vitro CQ susceptibility determination and interpretable *pfcrt *genotype. Among these, twenty-seven isolates appeared monoclonal on the fragment analysis method basis. CQ IC_50_s of selected isolates ranged from 5.36 nM to 219 nM (Table 1). Considering usual in vitro resistance CQ threshold, 21 isolates were susceptible (0–100 nM) and six were resistant (>100 nM). Nine isolates had IC_50 _values < 40 nM, seven isolates had IC_50 _values between 40 and 60 nM and 11 isolates had IC_50 _values > 60 nM.

### *Pfcrt, pfmdr1 *and putative transporter sequence analysis

**Figure 1:**
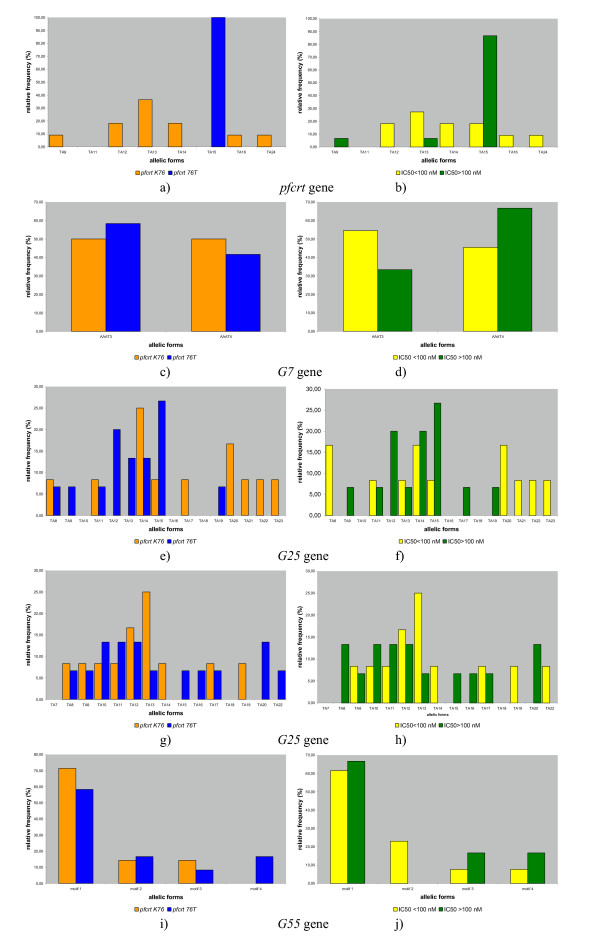
**Microsatellite allelic frequencies of *pfcrt *and putative transporter genes in 27 *P. falciparum *isolates according to *pfcrt *76 allele and chloroquine response phenotype. **Microsatellite allelic relative frequencies are given according to *pfcrt *K76 (wild allele, orange bars) or *pfcrt *K76T (mutant allele, blue bars) (Figure a, c, e, g and i), or according to inhibitory concentration values (IC_50_) for chloroquine (susceptible isolates, yellow bars; resistant isolates, green bars) (Figure b, d, f, h and j). The relative frequency of the various allelic forms within each haplotype subgroup is indicated. The microsatellites have either (AAAT)n alleles or (TA)n alleles and they are classified according to the number (n) of repeats. Figure a, b: *pfcrt *microsatellite in intron 4; Figure c, d: *G7 *microsatellite; Figure e, f: *G25 *microsatellite in intron 3; Figure g, h: *G25 *microsatellite in intron 4; and Figure i, j: *G55 *microsatellite. Motif 1: TAATATTATAATAT(TA)9T Motif 2: TAATAT(TA)11TMotif 3: TAATAT(TA)10T Motif 4: TAATAT(TA)9T.

Single nucleotide polymorphisms (SNPs) of *pfcrt *at positions 76 and 220 and *Pfmdr1 *at position 86, and variations in the microsatellite located nine bp downstream from A220S mutation in intron 4 of *pfcrt *were determined in 27 monoclonal isolates and in three reference strains (Table 3 and Figure 1a, 1b).

**Table 3:** Association between polymorphims in *pfmdr 1 *and 5 putative transporter genes and chloroquine response phenotype and *pfcrt 76 *mutant allele of 27 *P. falciparum *isolates.

Gene	Predicted product	Position	AA change	Nucleotide change	IC_50 _^a^CQ	*pfcrt *K76T
*pfcrt*	Transporter	76	K-T	AAA-ACA	P<.02	-
		220	A-S	GCC-TCC	P<.05	P<.001
*pfmdr1*	ABC transporter	86	N-Y	AAT-TAT	P<.05	P = .37
*G25*	Sulfate permease	Intron		G-A	NA	NA
*G30*	GTPase	Intron	-	C-G	P = .65	P = .9
*G49*	ABC/ATPase	146	Q-E	CAA-GAA	P = .11	P = .9
		1046	K-I	AAA-ATA	NA	NA
		1116	L-I	TTA-ATA	P = .54	P = .19
*G54*	Membrane protein	141	Y-Y	TAC-TAT	P = .58	P = .62
		144	T-T	ACG-ACA	P = .54	P = .63
*G70*	Choline transporter	105	E-K	GAA-AAA	P = .11	P = .9

^a^Fifty percent inhibitory concentration values : resistance value to chloroquine (CQ) corresponds to IC_50 _> 100 nM

NA for not applicable : no SNP was found in the studied isolates and the reference strains

SNPs of putative transporter genes in coding regions of *G49 *at positions 146, 1046 and 1116, of *G54 *at positions 141 and 144, of *G70 *at position 105, in intronic regions of *G25 *at position 2991 and of *G30 *at position 860 were determined in the same isolates as above. Variations in the microsatellite located in G7 at position 1390, in the microsatellites of G25 (one in intron 3 and one in intron 4), and in the microsatellite of G55 were also determined (Figure 1c–j).

### Correlation between transporter genotypes and in vitro CQ susceptibility

Polymorphism of *pfcrt *at positions 76 and 220 showed a significant association with chloroquine response (CQ response and K76T, A220S, *P *< .02, *P *< .05 respectively). Polymorphism of *pfmdr1 *at position 86 showed equally a significant association with in vitro chloroquine response (*P *< .05) (Table 3).

SNPs of putative transporter genes (*G7*, *G25*, *G30*, *G49*, *G54 *and *G70*) did not show association with CQR phenotype in our series of 27 isolates (Table 3).

The microsatellite located nine bp downstream from A220S mutation in intron 4 of *pfcrt *showed a (TAAA)3(TA)n polymorphism (Figure 1a, 1b). 1A single (TAAA)3(TA)15 microsatellite type was observed in all isolates having *pfcrt *76 mutant allele. Isolates having *pfcrt *76 wild allele showed various microsatellite types excepted the (TAAA)3(TA)15 type.

In *G7 *gene, at 1,390 locus, two microsatellite types were observed: (AAAT)3 and (AAAT)4 (Figure 1c, 1d). The microsatellite in intron 3 of *G25 *gene showed a (TA)n polymorphism with n ranging from eight to 23 (Figure 1e, 1f)). The microsatellite in intron 4 of *G25 *gene showed a (TA)n polymorphism with n ranging from eight to 22 (Figure 1g, 1h). The microsatellite G55 gene showed a (TAATAT(TA)nT) polymorphism with n ranging from 9 to 11 (Figure 1i, 1j). In all these putative transporter genes, no particular type of the studied microsatellites was significantly associated with *pfcrt *polymorphism or in vitro CQR.

## Discussion

It has been shown that *pfcrt *mutant parasites were globally present in endemic areas and that CQR mutant alleles could vary according to geographical origin [24-26]. Isolates originating from various African countries were included in the present study for two reasons: first, CQR is largely present and at comparable levels in the considered countries [27] and second, African parasite populations show less geographical variation in allele frequencies than Asian or South American populations [21]. Hence, risk of false associations in relation with parasite population structures bias appeared to be reduced.

The number of isolates included in the present study was relatively small in comparison of the number of falciparum malaria cases observed in the hospital parasitology laboratory during the study period. Travelers returning from Africa with a falciparum malaria attack harbor usually a mean of three circulating clones, with a range of one to 11 clones, according to previous results [22]. Only monoclonal isolates were included, which mostly limited the number of studied samples. The fragment analysis method used to enumerate the clones enabled us to work directly on monoclonal isolates instead of using culture-adapted cloned isolates. Thus potential bias in the IC_50_s determination was avoided.

In the present series, 15 isolates out of 27 had the *pfcrt *76 mutant genotype, though only six were classified as in vitro CQR (IC_50_s > 100 nM). These proportions were not surprising as it has previously shown that *pfcrt *K76T was found occasionally in isolates having CQ IC_50 _values located between 40 and 60 nM and almost constantly in isolates >60 nM [28]. These previous results were in favour of the hypothesis that CQR could result from a multi-gene process, *pfcrt *mutations being necessary but not sufficient to acquire CQR. The present study did not invalidate this hypothesis, but it failed to show the new putative transporter genes polymorphisms as associated with CQR. This could be due to the limited number of included isolates. However, an association between *pfcrt *and *pfmdr1 *and in vitro CQR was found in our series. In addition, these results were consistent with those of Anderson et al. who included numerous isolates (albeit originating from an unique geographic area) [21].

Microsatellite polymorphisms have been demonstrated as associated with resistant haplotypes [24]. Genome wide microsatellite scanning has shown marked linkage disequilibrium around *pfcrt *locus indicating four distinct founder events, with one ancestral mutant originating in Asia and subsequently invading Africa. Interestingly *Pfcrt *intron 4 contains a polymorphic (TAAA)n(TA)m microsatellite, with a particular type (TAAA)3(TA)15 found as strongly associated with *pfcrt *resistant allele in a previous study (data not shown) and also in the present series. No association was found between the microsatellite polymorphisms and the SNPs of the studied putative transporter genes or with in vitro CQR.

## Competing interests

The author(s) declare that they have no competing interests.

## Authors' contributions

SC performed the molecular analysis of the majority of the samples, performed the statistical analysis and contributed for the elaboration of manuscript. AN, DG and VH participated in the molecular typing of isolates and in the data analysis and helped to draft the manuscript. JLB participated in the parasite phenotyping and helped to draft the manuscript. RD participated in the design of the study and its coordination and contributed for the elaboration of manuscript. All authors read and approved the final manuscript.
